# Short-term effects of a combined nutraceutical of insulin-sensitivity, lipid level and indexes of liver steatosis: a double-blind, randomized, cross-over clinical trial

**DOI:** 10.1186/s12937-015-0019-y

**Published:** 2015-03-28

**Authors:** Arrigo FG Cicero, Martina Rosticci, Angelo Parini, Martino Morbini, Riccardo Urso, Elisa Grandi, Claudio Borghi

**Affiliations:** 1 Department of Medicine and Surgery Sciences, University of Bologna, Bologna, Italy; 2 Hypertension Research Center, Poliambulatorio Pad. 2, Via Albertoni, 15, 40138 Bologna, Italy

**Keywords:** Berberine, Chlorogenic acid, Nutraceutical, Insulin-sensitivity, Lipoproteins, Liver steatosis

## Abstract

**Background:**

Overweight subjects easily develop alterations of the glucose and lipid metabolism and are exposed to an increased cardiometabolic risk. This condition is potentially reversible through the improvement of dietary and behavioural habits. However, a well-assembled nutraceutical would be a useful tool to better improve the metabolic parameters associated to overweight and insulin resistance.

**Methods:**

To evaluate the effect of a combined nutraceutical containing berberine, chlorogenic acid and tocotrienols, we performed a double blind, cross-over designed trial versus placebo, in 40 overweight subjects with mixed hyperlipidaemia. After the first 8 weeks of treatment (or placebo), patients were asked to observe a 2-week washout period, and they were then assigned to the alternative treatment for a further period of 8 weeks. Clinical and laboratory data associated to hyperlipidaemia and insulin resistance have been obtained at the baseline, at the end of the first treatment period, after the washout, and again after the second treatment period.

**Results:**

Both groups experienced a significant improvement of anthropometric and biochemical parameters versus baseline. However, total cholesterol, LDL cholesterol, triglycerides, non-HDL cholesterol, fasting insulin, HOMA-IR, GOT and Lipid Accumulation Product decreased more significantly in the nutraceutical group versus placebo.

**Conclusions:**

This combination seems to improve a large number of metabolic and liver parameters on the short-term in overweight subjects. Further studies are needed to confirm these observations on the middle- and long-term.

## Background

Overweight and obesity are a worldwide leading health problem [1], especially when complicated by other diseases [2]. In particular, overweight is usually associated to a large number of metabolic changes, such as reduction of insulin-sensitivity, dyslipidaemia, accumulation of lipids in the liver, respectively predisposing to the development of type 2 diabetes, atherosclerosis and non-alcoholic steatohepatitis, all conditions increasing the risk to develop cardiovascular disease [3,4]. All these changes are potentially reversible with intensification of physical activity and improvement of dietary habits [5], while the efficacy/safety ratio of the available pharmaceutical drugs is not yet adequately defined [6].

Recent literature shows that a number of natural compounds seem to have positive effects on the metabolic changes associated to overweight [7,8]. Of particular interest seems to be soluble fibres, cinnamon, chromium picolinate, polyunsaturated fatty acids, berberine, chlorogenic acid, green tea, bitter gourd and banaba.

The main limitation in the clinical use of these products are the need to assume large quantities (for instance, cinnamon and bitter gourd), the scarce availability of clinical data (for instance, banaba), the relatively small metabolic effect (for instance, fibres and chromium), the need to use low doses in order to avoid side effects (for instance, berberine), and the cost (for instance, high quality green tea) [9]. On the other side, scarce data are available as it regards the clinical efficacy of combination of these substances.

In this context, the aim of our study was to evaluate the short-term metabolic effects of a combined nutraceutical containing berberine and chlorogenic acid on overweight subjects with mixed hyperlipidemia.

## Methods and materials

### Study design

This double blind, placebo controlled, randomized clinical trial was carried out in 40 moderately hypercholesterolemic subjects with metabolic syndrome, in primary prevention for cardiovascular diseases, consecutively enrolled in the ambulatory service of cardiovascular disease prevention in the Medical and Surgical Sciences Department of the University of Bologna.

The study consisted in a cross-over designed trial of 18 weeks, preceded by 4 weeks of diet stabilization in which patients received standard behavioural and qualitative (not quantitative) dietary suggestions to correct unhealthy habits. In particular, subjects were instructed to follow a general indication of a Mediterranean diet, avoiding excessive intake of dairy products and red meat derived products during the study, and maintaining overall constant dietary habits. Individuals were also generically encouraged to increase their physical activity by walking briskly for 20 to 30 minutes, 3 to 5 times per week, or by cycling.

Inclusion criteria were:

- age between 25 and 75
- LDL-Cholesterol (LDL-C) level between 130 and 190 mg/dl
- Body Mass Index (BMI) value between 25 and 30 kg/m^2^
- Triglycerides (TG) level between 150 and 400 mg/dl
- Fasting Plasma Glucose (FPG) level under 125 mg/dl.
- When present, assumption of hypolipidaemic or antihypertensive drugs stabilized since at least 6 months.

Exclusion criteria were:

- secondary prevention for cardiovascular diseases
- assumption of antidiabetic drugs
- inconstant or not-stabilized assumption of drugs which may interfere with lipid or glucose metabolism
- chronic gastrointestinal diseases and assumption of drugs for their treatment
- known thyroid, liver, renal or muscle diseases
- known allergy or intolerance to a component of the tested product
- any medical or surgical condition which could lead to an inconstant adhesion to the protocol.

The study was fully conducted in accordance with the Declaration of Helsinki, its protocol was approved by the Ethical Committee of the University of Bologna, and informed consent was obtained from all patients before the inclusion in the study.

### Treatments

After 4 weeks of diet and physical activity stabilization, patients were allocated to treatment with an indistinguishable pill of placebo or with an active product (Trixy®) containing Berberine from *Berberis aristata*, Tocotrienols form *Elaeis guineensis* and Chlorogenic acid form decaffeinated Green coffee (*Coffea canephora*), kindly offered by Nathura s.r.l. (Montecchio Emilia, RE).

After 8 weeks of treatment (or placebo), patients were asked to observe a 2-week wash-out period, and they were then assigned to the alternative treatment for a further period of 8 weeks. Clinical and laboratory data have been obtained at the baseline, at the end of the first treatment period, after the wash-out, and again after the second treatment period.

Randomization was done assigning an alphabetical code to each lot code (corresponding to treatment or placebo) impressed on the pillbox. Codes were then kept in a sealed envelope which was not opened until the end of the trial. Pillboxes were then mixed, and the investigators assigned a blinded pillbox to each enrolled patient.

Throughout the study, we instructed patients to take the product first dose on the day after they were given the study product. At the same time, all unused products were retrieved to the investigator.

### Assessments

Personal data, CVD history and pharmaceutical anamnesis of each patient were inquired at the beginning of the trial. Anthropometrical parameters, such as body weight, waist circumference and BMI were collected both at the beginning and during the trial, together with hemodynamic and biochemical parameters. BMI was calculated as weight in kilograms divided by the square of height in meters. Hemodynamic parameters were collected measuring orthostatic and clinostatic systolic and diastolic blood pressure (mean value of 3 interval measures). Also orthostatic and clinostatic wrist blood pressure (mean value of 3 interval measures) and cardiac frequency were collected.

All biochemical parameters were obtained after a 12-hour overnight fast. The following parameters were obtained or calculated through appropriate formulas: total cholesterol (TC) [10], high-density lipoprotein-cholesterol (HDL-C) [11], TG [12], LDL-C [13], non-HDL cholesterol (nonHDL-C), lipoprotein(a) (Lpa), FPG, fasting insulin, homeostatic model assessment of insulin resistance (HOMA-IR) [14], creatinine, Serum Uric Acid (SUA), liver transaminases (glutamic oxaloacetic transaminase, GOT; glutamate-pyruvate transaminase, GPT), Gamma Glutamyl-Transferase (γGT), Lipid Accumulation Product (LAP) and Hepatic Steatosis Index (HSI) [15]. All measurements were centrally performed in the laboratory of our department.

HOMA-IR is the simplest and most commonly used method to estimate insulin resistance. It was calculated as the product of basal glucose (mmol/l) and fasting insulin (μU/ml) divided by 22.5 [14].

LAP is calculated through the following formulas: (waist circumference (cm) - 65) × (TG (mmol/l)) for men and: (waist circumference (cm) - 58) × (TG (mmol/l)) for women, and is a useful marker of the individual cardiometabolic risk [16]. HSI, instead, is a simple index used to evaluate the presence or the severity of Non-alcoholic Fatty Liver Disease (NAFLD) through some biochemical parameters (TG, BMI, yGT, waist circumference) [16]. Adherence to behavioural counselling and to treatment/placebo, tolerability, acceptability and compliance were also assessed.

### Statistical analyses

The main outcome of the study was to evaluate the different effect of the active treatment and placebo on HOMA-index. Thus, we calculated the sample size of the study on the assumption that the HOMA-index standard deviation of the difference in the response variables is 0.7, so that with 40 patients enrolled, the probability was 80 percent that the study would have detected a treatment difference at a two-sided 0.05 significance level, if the true difference between treatments is 0.318 units.

Data have been analysed using intention to treat by mean of the Statistical Package for Social Science (SPSS) 21.0, version for Windows. Normally distributed baseline characteristics of the population have been compared using Student’s *t* test, non-normally distributed parameters using Mann–Whitney-*U* test. Two-way analyses of variance for crossover design were used to assess the effect of treatment during the assumption of placebo or treatment. All data are expressed as means and Standard Deviation (SD). To verify the basic assumptions of crossover design, besides the evaluation of period effect, the presence of a carryover effect was excluded. Level of statistical significance was set on *P* = 0.05.

## Results

The main characteristics of the enrolled patients have been resumed in Table 1. At the baseline the two treatment sequence group were balanced for all the investigated parameters. All the patients completed the study and no one experienced adverse events during the trial.

**Table 1:** **Baseline characteristics of the subjects enrolled in the trial**

N. 40	Mean	SD		Mean	SD
Age (years)	53.45	8.00	Total Cholesterol (mg/dL)	215.70	15.20
Weight (kg)	82.00	11.45	LDL-C (mg/dL)	132.15	17.50
Body Mass Index (kg/m2)	28.75	2.65	Triglycerides (mg/dL)	222.05	38.70
Waist Circumference (cm)	106.30	10.85	HDL-C (mg/dL)	39.10	3.40
SBP (mmHg)	135.50	14.90	Non HDL-C (mg/dL)	176.60	15.40
DBP (mmHg)	80.60	7.30	GOT (U/L)	24.90	10.10
Pulse Pressure (mmHg)	54.90	12.10	GPT (U/L)	28.10	12.95
MAP (mmHg)	98.95	8.75	Serum Uric Acid (mg/dL)	4.50	1.30
Heart Rate (bpm)	73.80	5.60	Creatinine (mg/dL)	8.95	1.25
FPG (mg/dL)	106.90	6.70	LAP	111.40	34.35
Insulin (μU/ml)	14.50	4.30	HSI	37.05	3.60
HOMA-IR	4.05	1.20			

Acronyms: SBP: systolic blood pressure, DBP: diastolic blood pressure, MAP: mean arterial pressure, LDL-C: LDL cholesterol, HDL-C: HDL cholesterol, Non HDL-C: non HDL cholesterol, FPG: fasting plasma glucose, HOMA-IR: homeostatic model assessment of insulin resistance, GOT: glutamic oxaloacetic transaminase, GPT: glutamate-pyruvate transaminase, LAP: lipid accumulation product, HSI: hepatic steatosis index.

Compared to baseline values, no change has been observed after both treatments as it regards Diastolic Blood Pressure (DBP), Pulse Pressure (PP), Heart Rate (HR), GOT, HSI, creatinine and SUA level.

Both groups experienced a significant decrease in body weight, BMI and waist circumference (p < 0.001), without significant difference between the treatments. Systolic and mean arterial pressure mildly, but significantly improved after diet-nutraceutical treatment. Lipid pattern improved after both treatments, however after diet-nutraceutical treatment TC, LDL-C, TG, and non-HDL-C decreased more significantly than after diet alone (p < 0.001). After diet-nutraceutical treatment HDL-C also mildly, but significantly, increased versus baseline. FPG similarly decreased after both treatments, but fasting insulin and HOMA significantly decreased versus baseline values after diet-nutraceutical treatment only: the reduction was also significant versus diet-placebo (p < 0.001 for both HOMA-IR and insulin). Then, GOT improved versus baseline and versus diet-placebo (p < 0.001) after diet- nutraceutical treatment. Finally, LAP improved after both treatments, but the reduction after diet-nutraceutical treatment was significantly higher than the one obtained with diet-placebo (p = 0.021) (Table 2).

**Table 2:** **Mean difference in changes of the studied parameters after placebo or nutraceutical treatment**

N.40	Mean difference in changes	*95% CI*		*P*
		*Lower*	*Upper*	
∆ Weight (kg)	−0,2	−0.9	1.1	0.534
∆ Body Mass Index (kg/m2)	−0,1	−0.8	1.0	0.687
∆ Waist Circumference (cm)	−0,6	−1.1	1.2	0.432
∆ Systolic Blood Pressure (mmHg)	0,2	−0.6	0.8	0.326
∆ Diastolic Blood Pressure (mmHg)	0,8	−0.1	1.3	0.091
∆ Pulse Pressure (mmHg)	−0,5	−0.9	0.2	0.084
∆ Mean Arterial Pressure (mmHg)	0,5	−0.2	0.9	0.102
∆ Heart Rate (bpm)	−0,1	−1.3	1.3	0.518
∆ Total Cholesterol (mg/dL)	−19,5	−61.4	−9.3	<0.001
∆ LDL-C (mg/dL)	−15,4	−45.3	−2.7	<0.001
∆ Triglycerides (mg/dL)	−21,4	−64.3	−1.1	<0.001
∆ HDL-C (mg/dL)	0,3	−0.1	2.1	0.054
∆ Non HDL-C (mg/dL)	−19,7	−62.2	−3.0	<0.001
∆ Fasting Plasma Glucose (mg/dL)	−0,1	−9.4	7.6	0.433
∆ Insulin (μU/ml)	−1	−1.1	−0.1	0.018
∆ HOMA-IR	−0,3	−0.5	−0.1	<0.001
∆ GOT (U/L)	−0,3	−0.7	0.9	0.681
∆ GPT (U/L)	−7,8	−11.4	−0.9	0.027
∆ Serum Uric Acid (mg/dL)	−2,4	−3.7	1.4	0.349
∆ Creatinine (mg/dL)	−0,5	−0.9	0.8	0.441
∆ Lipid Accumulation Product	−10,4	−44.5	−0.3	0.002
∆ Hepatic Steatosis Index	1,3	−0.1	2.9	0.068

Acronyms: LDL-C: LDL cholesterol, HDL-C: HDL cholesterol, Non HDL-C: non HDL cholesterol, HOMA-IR: homeostatic model assessment of insulin resistance, GOT: glutamic oxaloacetic transaminase, GPT: glutamate-pyruvate transaminase.

The wash-out did not determine a significant carry-over effect in both group, probably because its relatively short duration and because the improvement in dietary habits persisted during this phase. However, the group who began with diet-nutraceutical treatment, at the end of the study experience a regression of a large number of metabolic parameters (HOMA-index, TC, LDL-C, non HDL-C) to values no more significantly different than the baseline ones (p > 0.05 for all).

## Discussion

The insulin-sensitizing property of coffee [15] has been partly attributed to its content in chlorogenic acid, an ester formed from cinnamic acids and quinic acid. In particular, chlorogenic acid seems to act as insulin sensitizer that potentiates insulin action similar to the therapeutic action of metformin [17]. However, recent data suggest that it could also exert its antidiabetic action by inhibiting α-amylase and α-glucosidase activities [18].

Moreover, the effect of chlorogenic acid on expression of hepatic peroxisome proliferator-activated receptor-alpha [19] and on activation of 5′ AMP-activated protein kinase [20] partly explain the presumed liver protective effect of coffee from non-alcoholic fatty liver disease [21].

Berberine has a well-known antihyperlipidaemic effect, both inhibiting the liver LDL-receptor inactivation by inhibiting PCSK9 and activating the 5′ AMP-activated protein kinase [22]. Moreover, berberine exerts insulin-sensitizing effects with a metformin-like mechanism of action [23]. Recent data also suggest that berberine could also induce improvement in liver steatosis, probably as consequence of the relevant changes induced in triglycerides level and insulin-sensitivity [24].

So, as expected from the available literature data (16–24), after a combined treatment with berberine and chlorogenic acid we observed a significant improvement in LDL-C (−24%), TG (−19%), non HDL-C (−22%), fasting insulin (−5%) and HOMA index (−10%). Moreover, we also observed a significant improvement in LAP (−25%), a validated index of liver steatosis [16].

All these effects could be particularly relevant in the management of overweight subjects complicated with metabolic syndrome and/or non-alcoholic fatty liver disease, both condition associated to high cardiometabolic disease risk [25,26] and whose management is actually mainly based on diet and life-style improvement [27,28].

This study has some limitation. The first one is the relatively small sample size, partially balanced by the cross-over design of the trial. The second one is the relatively short duration of the trial, however comparable with the most part of explorative trials carried out with metabolically active nutraceuticals. Then, we have not prescribed a specific diet based on bromatological data, but we gave only general dietary suggestion finalized at avoiding dietary excess in order to simulate more strictly a condition of general practice. Finally, we have not directly evaluated the degree of liver steatosis. On the other side, validated index of liver steatosis have been used, supported by recent literature. Finally, we did not compare the effects of berberine and chlorogenic acid alone and with their association, but the effect of the two components alone on the parameters investigated was already known in literature.

## Conclusions

On the basis of our preliminary data, a nutraceutical containing berberine and chlorogenic acid seems to be well-tolerated and able to improve a large number of metabolic and liver parameters on the short-term in overweight patients with mixed hyperlipidaemia. Further studies are needed to confirm these observations on the middle- and long-term.

## Abbreviations

BMI: Body mass index
DBP: Diastolic blood pressure
FPG: Fasting plasma glucose
γGT: Gamma glutamyl-transferase
GOT: Glutamic oxaloacetic transaminase
GPT: Glutamate-pyruvate transaminase
HDL-C: High-density lipoprotein-cholesterol
HOMA-IR: HOmeostatic model assessment of insulin resistance
HR: Heart rate
HSI: Hepatic steatosis index
LAP: Lipid accumulation product
LDL-C: Low-density lipoprotein-cholesterol
Lpa: Lipoprotein(a)
MAP: Mean arterial pressure
NAFLD: Non-alcoholic fatty liver disease
nonHDL-C: nonhigh-density lipoprotein-cholesterol
PP: Pulse pressure
SBP: Systolic blood pressure
SD: Standard deviation
SUA: Serum uric acid
TC: Total cholesterol
TG: Triglycerides
WC: Waist circumference
